# Multi-Reference Geometric Risk and Sparse Depth Refinement for Streaming 3D Reconstruction

**DOI:** 10.3390/s26216995

**Published:** 2026-11-05

**Authors:** Chunguang Wang, Wei Sun, Jianlong Zheng, Meijing Ge, Yuming Qi

**Affiliations:** 1 School of Mechanical Engineering, Tianjin Sino-German University of Applied Sciences, Tianjin 300350, China; 2 Institute of Robotics and Intelligent Equipment, Tianjin University of Technology and Education, Tianjin 300222, China

**Keywords:** streaming 3D reconstruction, geometric risk, sparse depth, geometric anchoring, budget-aware intervention, depth recovery, RGB-D

## Abstract

**Highlights:**

**What are the main findings?**

- A training-free multi-reference geometric risk achieves a median Spearman rank correlation of 0.635 with scale-aligned depth error across 11 public scenes and a correlation of 0.556 on the real-robot Sequence E.
- With identical 2% sparse depth anchors, conservative local recovery reduces Sequence E held-out AbsRel by 40.69% relative to Sparse Global; consistent depth improvements are observed across 11 public scenes under the evaluated protocol.

**What are the implications of the main findings?**

- Sparse geometric anchoring supports conservative refinement of selected depth predictions from limited registered depth samples, without retraining the backbone or changing its camera poses and streaming state.
- Multi-reference geometric consistency offers an interpretable, training-free frame-quality signal that complements model-native confidence and supports the analysis of selective geometric intervention.

**Abstract:**

Streaming feed-forward 3D reconstruction produces depth predictions at successive frames, but cross-view geometric consistency and local depth accuracy may vary. We present a training-free, output-level approach that combines Multi-Reference Geometric Risk assessment with global-referenced conservative sparse depth refinement, leaving the pretrained backbone, its predicted poses, and its streaming state unchanged. Risk derived from predicted depth, intrinsics, and poses achieves a median Spearman correlation of 0.635 with scale-aligned depth error across 11 public scenes and a correlation of 0.556 on the real-robot Sequence E. With matched 2% sparse depth anchors, local refinement reduces Sequence E held-out AbsRel by 40.69% relative to Sparse Global. Spatial refinement also improves mean held-out depth error in all 55 public scene–policy comparisons and all 200 predefined fixed-mask scene–policy–seed comparisons. Retrospective risk-based frame ranking provides diagnostic benefits under reference-scale normalization, but the observed allocation gains are protocol-dependent and do not establish consistent improvements under historical sparse-scale propagation or deployable online scheduling. In an independent Bonn Leica comparison using shared ground-truth camera poses, Spatial increases recall at a 5 cm threshold but has lower precision and F-score than Sparse Global. These findings establish the value of conservative sparse depth refinement and multi-reference risk as complementary geometric-quality evidence, while distinguishing depth accuracy from downstream 3D geometric performance.

## 1. Introduction

Classical visual SLAM systems, including ORB-SLAM2 and DynaSLAM, use explicit tracking and optimization for mapping and dynamic-scene robustness [1,2]. Feed-forward alternatives such as DUSt3R and MASt3R instead infer geometric representations directly from images [3,4].

Streaming approaches extend this paradigm through spatial memory or recurrent state (Spann3R and CUT3R [5,6]), large-scale multi-view prediction (Fast3R and VGGT [7,8]), and dynamic or temporally aligned reconstruction (MonST3R, Align3R, and MASt3R-SLAM [9,10,11]). LingBot-Map supports long sequences through historical geometric context [12], with motion-aware context adaptation studied in [13]. E3D-Bench documents remaining scene-dependent performance variation [14].

However, continuous prediction does not ensure reliable depth across frames: motion, occlusion, pose errors, and depth–translation scale mismatch may all affect cross-view agreement. Test3R and TTT3R adapt models at test time [15,16], Trust3R models point-level uncertainty [17], and native-confidence calibration remains imperfect [18]. We instead examine a training-free frame-level consistency signal without changing the backbone.

Generalized monocular depth priors, including Depth Anything and Metric3D [19,20], address prediction and metric-depth generalization rather than sparse external measurement access. Sparse-to-Dense and geometric-prior/scale-adaptation approaches [21,22,23,24,25] instead illustrate how sparse or auxiliary geometric constraints can guide depth correction. Unlike active viewpoint or within-frame measurement selection [26,27], our study compares retrospective access to registered depth on selected frames of a fixed sequence.

We address frame-level risk assessment and conservative sparse depth correction using a frozen LingBot-Map backbone [12]. Predicted depth, intrinsics, and poses yield Multi-Reference Geometric Risk; 2% registered-depth anchors support local multiplicative corrections subject to global-scale fallback. We additionally compare retrospective frame-priority policies under separate reference-normalized and historical-scale protocols. The backbone weights, camera poses, and streaming state remain unchanged; we do not claim causal online sensing.

We evaluate risk ranking on 11 public scenes and the mobile-robot Sequence E, matched sparse recovery, retrospective allocation, and a shared-pose Bonn Leica geometry comparison. Across the 11 public scenes, the median Spearman correlation between risk and scale-aligned depth error is 0.635; on Sequence E, the corresponding correlation is 0.556. With identical 2% anchors, local recovery reduces Sequence E held-out AbsRel by 40.69% versus Sparse Global. The independent Leica evaluation reveals a geometry accuracy–completeness trade-off.

1. We develop Multi-Reference Geometric Risk as a training-free frame-level diagnostic based on the frozen backbone’s depth, camera intrinsics, and poses. By comparing two historical viewpoints after scale-aligned reprojection, it provides complementary evidence for ranking scale-aligned depth error without learning a separate uncertainty estimator.
2. We develop global-referenced conservative local depth refinement from sparse metric anchors. A robust frame-level scale provides the fallback, while locally supported multiplicative corrections are accepted only under a predefined local/global consistency condition. This design isolates spatial refinement from a single-scale correction under matched sparse measurements.
3. We evaluate the two contributions separately and jointly: multi-scene risk–error ranking, matched Sparse Global versus Spatial depth recovery, repeated sparse-anchor sampling, and retrospective frame-priority comparisons under explicit depth-access rules. A single-scene shared-pose Leica comparison reports independent 3D geometry metrics and their accuracy–completeness trade-off without treating frame allocation or 3D gains as universally superior.

## 2. Related Work

### 2.1. Streaming and Feed-Forward 3D Reconstruction

DUSt3R and MASt3R infer image-based geometric representations [3,4], while Fast3R and VGGT extend feed-forward inference to larger multi-view inputs [7,8].

Spann3R and CUT3R incorporate historical spatial memory or persistent state [5,6]. MonST3R and Align3R address dynamic geometry and temporal depth alignment [9,10]; MASt3R-SLAM incorporates feed-forward priors into dense SLAM [11].

LingBot-Map provides the streaming reconstruction backbone for this study. It combines geometric context, anchor context, a pose-reference window, and trajectory memory to support long input sequences [12]. Motion-Aware Geometric Context Adaptation alters the historical context used by LingBot-Map through motion-dependent keyframe and pose-window decisions [13]. In contrast, our approach holds the prediction backbone and its streaming state fixed, evaluates the geometric reliability of its outputs, and refines selected depth maps with externally sampled sparse observations. E3D-Bench documents scene-dependent performance variation among geometric foundation models [14]. Our emphasis is therefore output-level geometric risk assessment and conservative sparse depth refinement, rather than a new reconstruction network or a replacement for model-native confidence.

### 2.2. Geometric Reliability, Confidence, and Test-Time Adaptation

As feed-forward 3D reconstruction moves toward longer sequences and more complex scenes, estimating and improving geometric reliability at test time has emerged as a distinct research problem. One line of work directly adapts model outputs or internal representations. Test3R constructs test-time supervision from geometric consistency among inputs and updates the model to improve reconstruction consistency [15]. TTT3R targets continuous reconstruction and uses alignment information between current observations and historical representations to adapt the test-time state when sequences exceed the original training context [16]. In both cases, reliability or consistency evidence is ultimately used to modify the model or its internal representation.

A second line explicitly estimates predictive reliability. Trust3R introduces evidential uncertainty modeling for feed-forward reconstruction and represents geometric reliability at the point level; it evaluates the resulting uncertainty using tools such as sparsification and risk-coverage analysis [17]. Complementary calibration analysis of existing feed-forward reconstruction models shows that model-native confidence can provide useful error ranking, while cautioning that ranking performance does not imply that the confidence value itself is calibrated to the true error magnitude [18].

Our risk aggregates backbone-derived cross-view inconsistency at frame level. It evaluates error ranking and retrospective intervention priority, not calibrated error or guaranteed recovery utility.

### 2.3. Sparse Geometric Guidance and Resource-Aware Intervention

Sparse-to-Dense demonstrates sparse-depth-constrained prediction [21]; Depth Anything and Metric3D address depth generalization [19,20].

Pow3R, zero-shot depth completion, and test-time geometric prompting incorporate geometric priors or sparse measurements [22,23,24].

Region-aware Depth Scale Adaptation estimates region-wise scale and shift from sparse measurements and expands support neighborhoods before fitting local surfaces [25]. Our sparse recovery instead employs fixed image blocks, multiplicative scale only, a frame-wide reference scale, and an explicit local-support and scale-consistency fallback. We do not claim that sparse depth calibration itself is new; the contribution lies in a compact, conservative correction for frozen streaming predictions and in directly isolating its benefit from global-only scaling at matched anchor locations.

Resource-constrained measurement provides an additional experimental setting. AREA3D selects future views for active reconstruction [26], whereas ProbeMDE selects informative sparse measurements within an image [27]. Here the video sequence is given, the backbone predictions are frozen, and sparse depth locations are sampled rather than actively optimized. A frame-level access budget specifies which already predicted frames may use simulated external depth samples; retrospective rankings compare prioritization signals without evaluating a causal acquisition controller. Table 1 contrasts these decision levels and the corresponding intervention mechanisms.

## 3. Methodology

### 3.1. Problem Formulation and Overall Framework

For a frozen streaming backbone, frame t produces predicted depth D_t_, intrinsics K_t_, and camera-to-world pose T_t_ from RGB and its prior state, as formalized in Equation (1).

(1) (*D_t_*,*K_t_*,*T_t_*) = *F_θ_*(*I_t_*,*H_t_*_−1_)

In Equation (1), *I_t_* is the input RGB image; *F_θ_* is the pretrained mapping with fixed parameters *θ*, and *H_t_*_−1_ is its historical state. The outputs are *D_t_* (predicted depth), *K_t_* (intrinsics), and *T_t_* = (*R_t_*, *t_t_*), a camera-to-world (C2W) pose with rotation *R_t_* and camera origin *t_t_*. Only these outputs enter risk computation.

The risk score ranks cross-view inconsistency rather than calibrated error. A normalized retrospective budget B selects eligible frames for sparse registered-depth intervention; no online sensor activation is evaluated.

Selected frames receive conservative local refinement D_t_* from registered sparse depth; unselected frames retain D_t_ under the direct output rule. The historical-scale benchmark has a separate, explicitly defined carried-scale rule.

**Figure 1:**
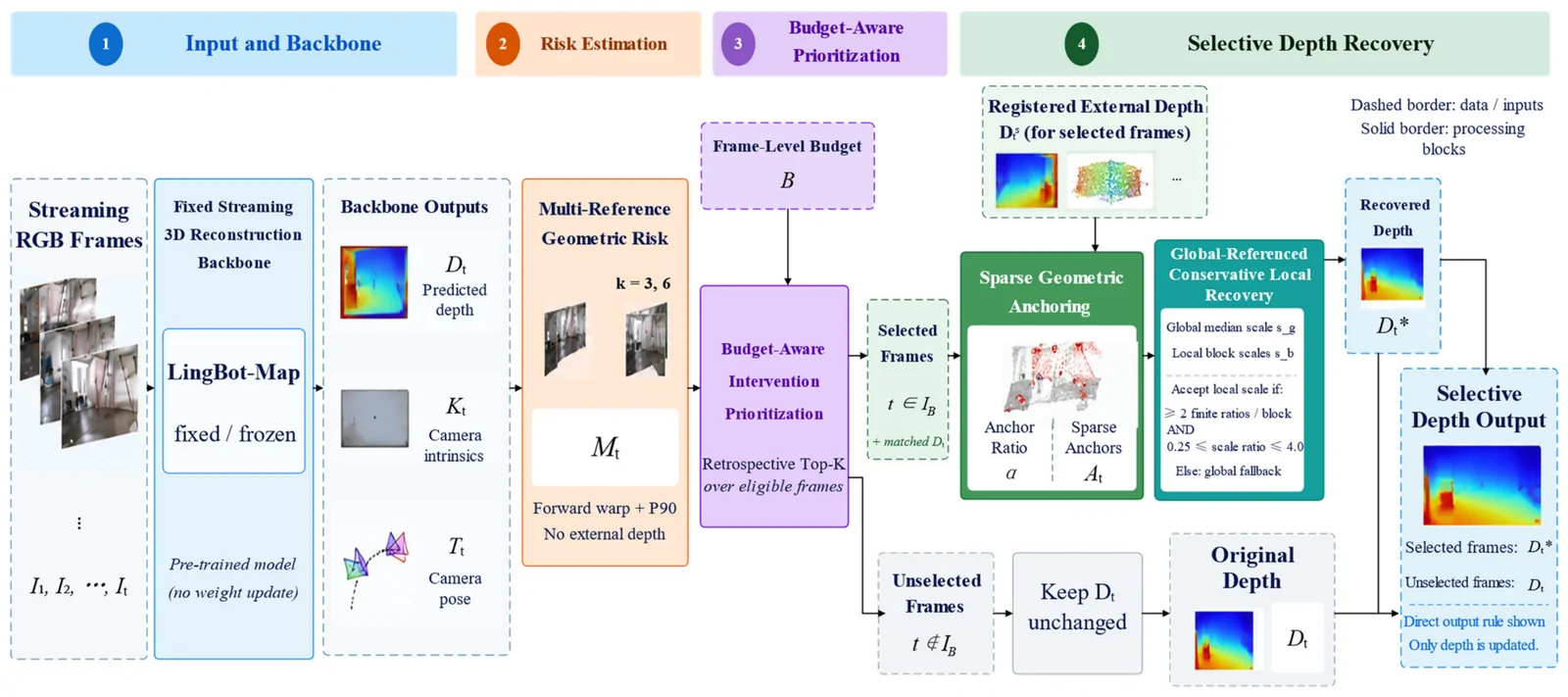
Overall architecture of the training-free, output-level intervention framework. The frozen streaming backbone supplies predicted depth, intrinsics, and camera pose for multi-reference risk analysis. A retrospective fixed-budget policy selects frames for 2% Sparse External-Depth Anchoring and Global-Referenced Local Recovery. Registered external depth enters only the selected-frame recovery branch; the backbone and risk estimator remain unchanged. The superscript * in Dₜ* denotes the refined depth map.

The complete pipeline consists of four consecutive modules: Multi-Reference Geometric Risk → Budget-Aware Intervention Prioritization → Sparse Geometric Anchoring → Global-Referenced Conservative Local Recovery. The overall framework is illustrated in Figure 1.

### 3.2. Multi-Reference Geometric Risk Estimation

**Figure 2:**
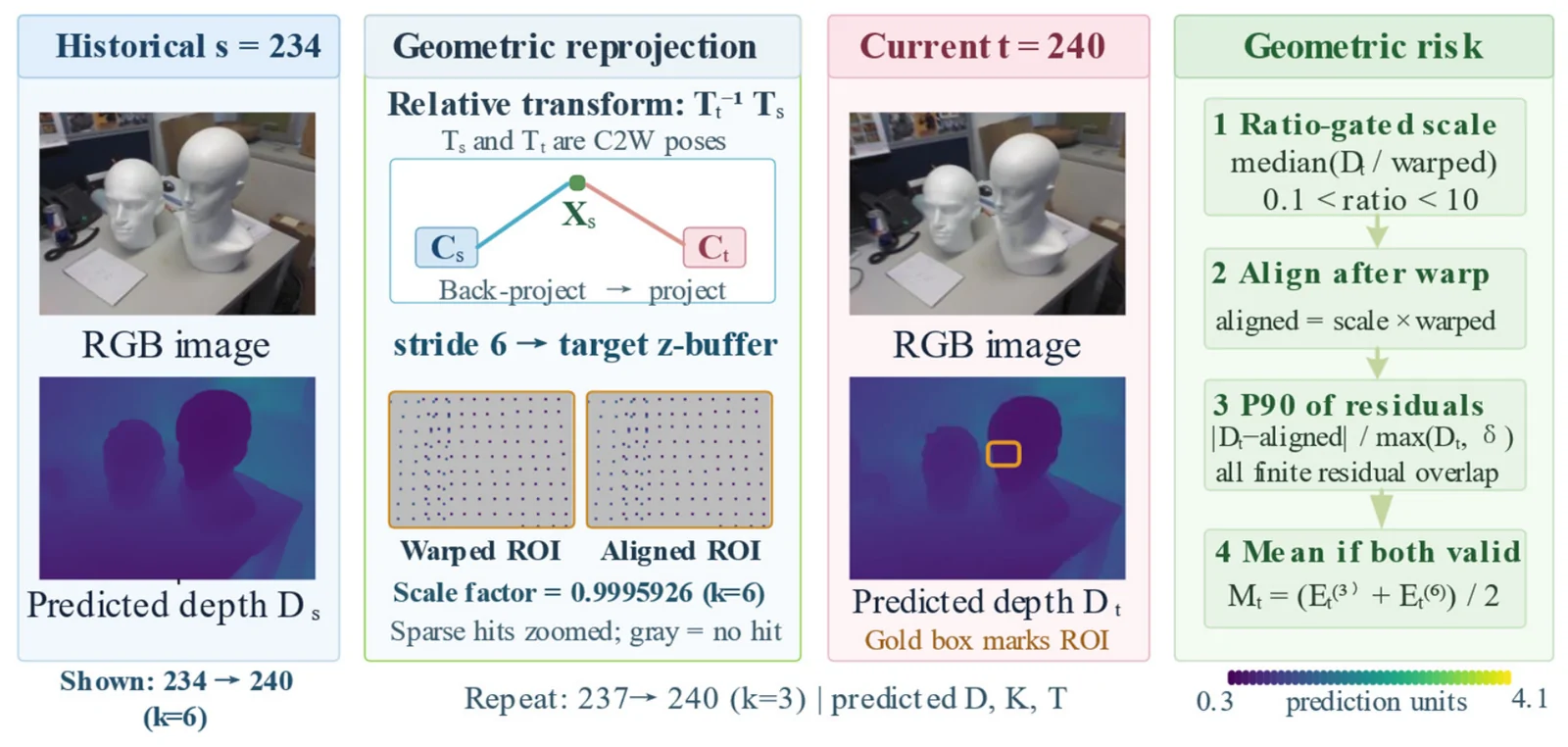
Geometry-based multi-reference risk estimation. Predictions at historical frames s = t − 3 and t − 6 are independently transformed into the current view using the exported camera-to-world poses and a target-view z-buffer. Median ratio alignment is estimated on the ratio-gated overlap; P90 residuals are subsequently calculated over the full finite overlap. The two valid lag scores are averaged to form M_t_.

Multi-Reference Geometric Risk compares current depth with two historical predictions after forward reprojection and scale alignment, using only backbone depths, intrinsics, and poses. The procedure is illustrated in Figure 2.

For current frame t, the two history offsets are k ∈ {3, 6}, and each source frame is *s* = *t* − *k*. Thus, lag 3 and lag 6 mean three and six prediction indices before *t*, not fixed elapsed times. Let *u_s_* = (*x_s_*, *y_s_*)*^T^* denote a source pixel, and let *ũ_s_* = (*x_s_*, *y_s_*, 1)*^T^* be its homogeneous image coordinate. The source camera point is *p_s_^C^* = *D_s_*(*u_s_*)*K_s_*^−1^*ũ_s_*. With C2W poses *T_s_* = (*R_s_*, *t_s_*) and *T_t_* = (*R_t_*, *t_t_*), the source point is mapped to the current camera as *p^C^_s→t_* = *R_t_^T^*(*R_s_ p_s_^C^* + *t_s_* − *t_t_*); target image coordinates are obtained by projection through K_t_ and perspective division. Equation (2) summarizes this transformation, while the depth coordinate of *p^C^_s→t_* defines the forward-warped depth. Reprojection uses camera-Z depth and assumes that predicted depth and camera translations have compatible scale units; otherwise, projected pixel correspondences may be displaced, and subsequent post-warp scale alignment cannot restore them.

(2) $$p_{s\to t}^{C}=R_{t}^{T}\left[R_{s}\left(D_{s}(u_{s})K_{s}^{-1}{\tilde{u}}_{s}\right)+t_{s}-t_{t}\right],\;{\tilde{u}}_{t}\sim K_{t}p_{s\to t}^{C}$$

Projected samples outside the image or behind the target camera are discarded. Nearest-positive-depth z-buffering resolves collisions. Historical depth is sampled at stride 6 before warping.

For each lag *k*, let $V_{t,\mathrm{res}}^{\left(k\right)}$ denote the target pixels for which the current depth is finite and positive, a valid positive historical depth has been forward-warped, and the resulting relative residual is finite. Let $V_{t,\mathrm{scale}}^{\left(k\right)}$ be its ratio-gated subset, requiring finite current-to-warped depth ratios strictly between 0.1 and 10. Equation (3) takes the median of these ratios over $V_{t,\mathrm{scale}}^{\left(k\right)}$. This robust gate applies to scale estimation only, not to the subsequent P90 residual domain.

(3) $$s_{t}^{\left(k\right)}=\underset{u\in V_{t,\mathrm{scale}}^{\left(k\right)}}{\mathrm{median}}\frac{D_{t}(u)}{D_{t-k\to t}(u)}$$

After median scale alignment, Equation (4) computes an absolute relative residual normalized by max(*D_t_*, *δ*). P90 uses the full finite-residual overlap, not only the ratio-gated subset used to estimate scale.

(4) $$\begin{array}{l} e_{t}^{\left(k\right)}(u)=\frac{\left|D_{t}(u)-s_{t}^{\left(k\right)}D_{\left(t-k\right)\to t}(u)\right|}{\max\left(D_{t}(u),\delta \right)},\\ E_{t}^{\left(k\right)}=P_{90}\left(\left\{e_{t}^{\left(k\right)}(u)|u\in V_{t,\mathrm{res}}^{\left(k\right)}\right\}\right) \end{array}$$

Here *δ* = 10^−6^ safeguards division. P90 summarizes large residuals; this scale alignment cannot repair pixel correspondences displaced by depth–translation gauge mismatch.

(5) $$M_{t}=\frac{E_{t}^{\left(3\right)}+E_{t}^{\left(6\right)}}{2}$$

The final risk *M_t_* averages valid lag-3 and lag-6 P90 scores. Each lag requires at least 100 sampled source points, warped overlaps, scale samples, and finite residuals; if either lag fails, *M_t_* is undefined rather than imputed.

### 3.3. Budget-Aware Intervention Prioritization

Let T_M_ denote the set of frames on which both lag-based risks are valid. For a fixed eligible evaluation window and normalized frame-query budget *B*, Equation (6) defines the allowed number of measurements *K_B_* and the corresponding retrospective Top-K subset I_B_. The within-frame anchor fraction α is a separate quantity: *K_B_* counts queried frames, while α controls sparse depth samples within each selected frame. A full-window ranking needs all candidate risk values and therefore does not establish a future-blind acquisition policy.

(6) $$K_{B}=\left\{\begin{array}{ll} \max\left(1,\mathrm{round}\left(B|T_{M}|\right)\right), & |T_{M}|>0,\\ 0, & |T_{M}|=0, \end{array}\right.\;I_{B}=\underset{t\in T_{M}}{\mathrm{TopK}}\left(M_{t},K_{B}\right)$$

In the direct output rule, unselected frames retain *D_t_* and selected frames receive sparse recovery. A separate historical-scale evaluation carries the last measured sparse global scale to unselected outputs.

Each M_t_ is computed from the current predicted depth, intrinsics, and pose, together with historical backbone outputs at lags 3 and 6; it requires no future observations. However, full-window Top-K allocation uses later risk scores to rank earlier frames. This is retrospective selection rather than a causal online trigger.

### 3.4. Sparse Geometric Anchoring and Conservative Local Recovery

For a selected frame t ∈ I_B_, let Dts be the external depth that has been geometrically registered to the predicted image grid. “External depth” denotes depth information separate from the RGB-only backbone input; in the reported Sequence E experiments it is simulated by sparsely sampling the continuously recorded registered D435i depth. Thus, the budget limits experimental depth-sample access and not measured camera acquisition, USB transmission, or power use. No dense sensor depth is supplied to the recovery operator on an unselected frame.

Let $\Omega_{t}^{\mathrm{valid}}$ be the image pixels at which *D_t_* and the registered external depth *D_ts_* are both finite, positive, spatially corresponding, and pass the fixed depth-validity rules. The set At contains N_a_ uniformly sampled anchors (*u_i_*, *d_i_^s^*), where *d_i_^s^* = *D_t_^s^*(*u_i_*) and *N_a_* is determined by α = 2% of |$\Omega_{t}^{\mathrm{valid}}$| according to Equation (7). Sampling is without replacement; the evaluation mask excludes these observed anchor pixels. A frame query and a pixel-level anchor count are separate budget units.

(7) $$N_{a}=\max\left(1,\mathrm{round}\left(\alpha \left|\Omega_{t}^{\mathrm{valid}}\right|\right)\right),\;A_{t}={\left\{\left(u_{i},d_{i}^{s}\right)\right\}}_{i=1}^{N_{a}}$$

If the valid sampling domain is empty, no anchors are selected (*N_a_* = 0), recovery is skipped, and the original predicted depth is retained. Equation (7) applies to a nonempty valid sampling domain. For anchor (*u_i_*, *d_i_^s^*) ∈ At, the ratio *ρ_i_* = *d_i_^s^*/max(*D_t_*(*u_i_*), *δ*) maps the model-predicted depth toward the registered sensor-depth scale. The median of the valid ratios gives global factor s_g_ in Equation (8). All 32 × 32 local blocks use that same *s_g_* as their conservative fallback, so introducing local correction does not require any additional depth anchors relative to Sparse Global. A queried frame counts against the fixed frame budget even if insufficient finite anchor ratios subsequently cause recovery to be skipped.

(8) $$\rho_{i}=\frac{d_{i}^{s}}{\max\left(D_{t}(u_{i}),\delta \right)},\;s_{g}=\underset{(u_{i},d_{i}^{s})\in A_{t}}{\mathrm{median}}\rho_{i}$$

If fewer than ten finite anchor ratios exist, recovery is skipped and *D_t_* retained. Otherwise *s_g_D_t_* is both the Global baseline and the fallback for unsupported blocks.

The depth grid is partitioned into non-overlapping 32 × 32 pixel blocks. For each block *b*, *A_t_,b* is the subset of available anchor pairs with pixels in *b*; *s_b_* is the median valid anchor ratio for that block and *r_b_* = *s_b_*/max(*s_g_*, *δ*) is its scale ratio relative to the global reference, as defined in Equation (9). These are multiplicative depth corrections; the method does not estimate a new camera pose or learn a spatial deformation field. In Equation (9), the anchor set is the current-frame set *A_t_* defined in Equation (7); *A_t_,_b_* consists only of its anchor pairs with pixel coordinates in block b.

(9) $$\begin{array}{l} A_{t,b}=\left\{\left(u_{i},d_{i}^{s}\right)\in A_{t}|u_{i}\in b\right\},\\ s_{b}=\underset{\left(u_{i},d_{i}^{s}\right)\in A_{t,b}}{\mathrm{median}}\rho_{i},\;r_{b}=\frac{s_{b}}{\max\left(s_{g},\delta \right)} \end{array}$$

Equation (10) accepts *s_b_D_t_* only if |*A_t_*,*b*| ≥ 2 and 0.25 ≤ *r_b_* ≤ 4.0; every other block uses *s_g_D_t_*. Invalid or insufficient local evidence therefore never forces a local correction.

(10) $$D_{t}^{*}(u)=\left\{\begin{array}{ll} s_{b}D_{t}(u), & u\in b,\;|A_{t,b}|\geq 2,\;0.25\leq r_{b}\leq 4.0,\\ s_{g}D_{t}(u), & \mathrm{otherwise}. \end{array}\right.$$

**Figure 3:**
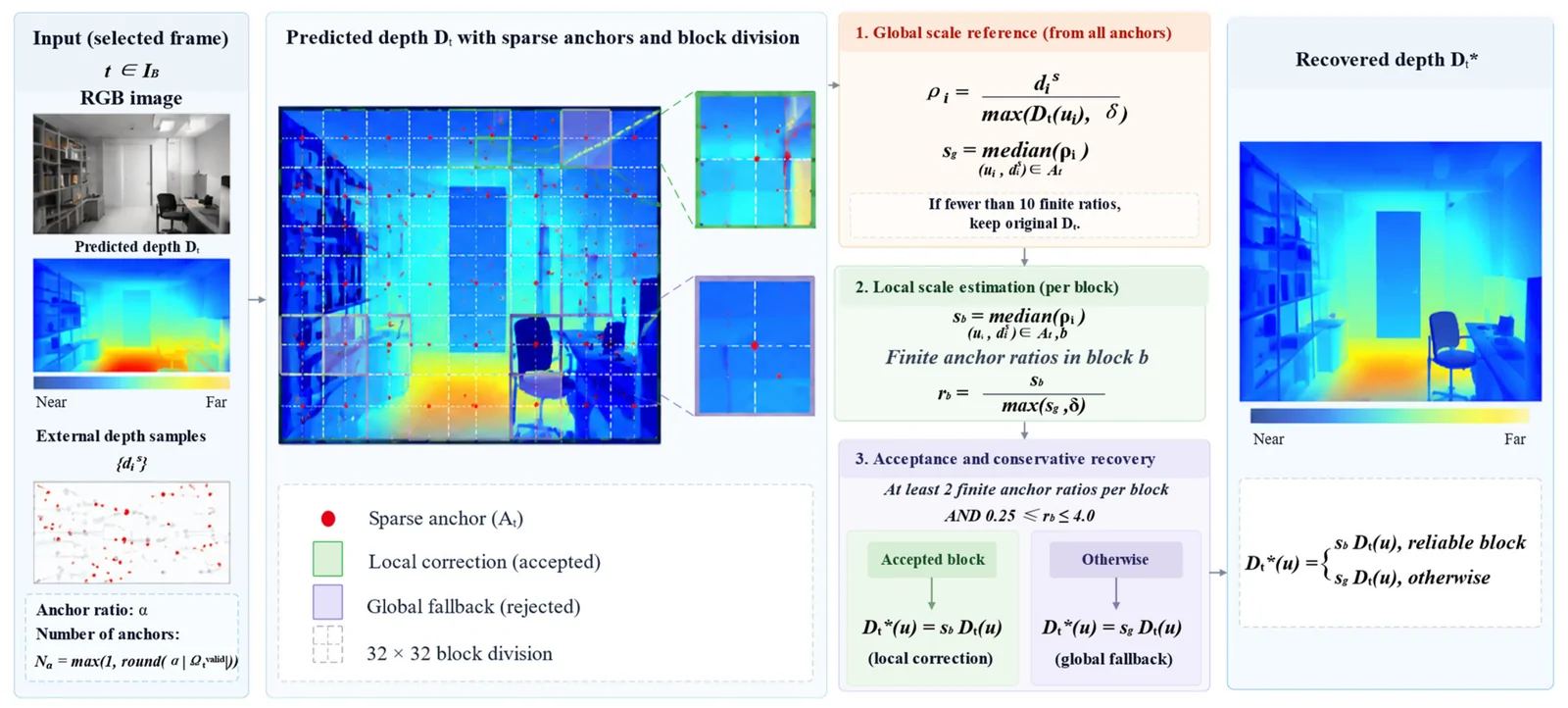
Sparse Geometric Anchoring and Global-Referenced Conservative Local Recovery. The superscript * in Dₜ* denotes the refined depth map.

Unlike flexible scale-and-shift or surface fitting [25], recovery uses fixed blocks and multiplicative scales with a global fallback, without optimizing the backbone. The sparse anchoring and conservative local recovery procedure is illustrated in Figure 3.

### 3.5. Computational Characteristics and Method Boundaries

For image size H × W and fixed lags {3, 6}, forward warping and finite-overlap statistics have O(HW) complexity per frame, with sampling stride 6.

Recovery uses anchor ratios, medians, and fixed-block traversal only on selected frames. Neither module trains an auxiliary network or updates backbone poses or state. Measured CPU component costs are reported in Section Historical Sparse-Scale Propagation and Matched Recovery Evaluation, but complete inference latency is not assessed.

## 4. Experimental Setup and Evaluation Protocol

### 4.1. Datasets, Experimental Platform, and Setup

RQ1 tests error ranking; RQ2 compares local and global correction using identical sparse anchors and examines one Leica geometry sequence; RQ3 compares retrospective allocation under reference-normalized and historical-scale protocols.

We evaluate frozen LingBot-Map outputs [12] on TUM RGB-D [28], Bonn Dynamic RGB-D [29], 7-Scenes [30], and a captured indoor robot sequence (Sequence E).

The 11 public scenes comprise TUM walking_rpy, walking_xyz, sitting_static, and sitting_xyz; Bonn person_tracking, crowd, moving_box, and static_close_far; and 7-Scenes Heads and Stairs seq01/seq04.

**Figure 4:**
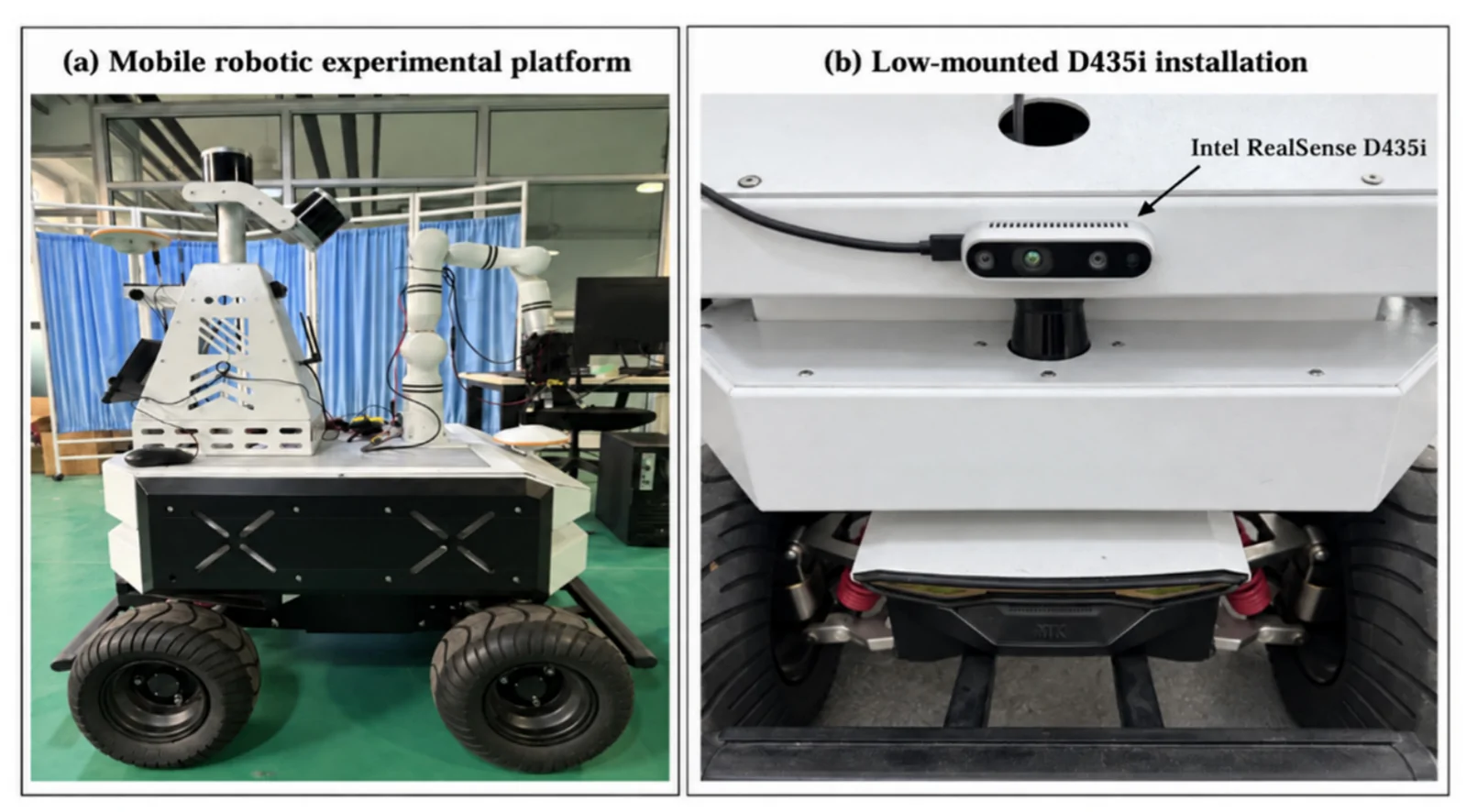
Mobile robotic experimental platform and the RGB-D sensing device used in this study.

Sequence E was recorded with a mobile robot and a registered Intel RealSense D435i RGB-D camera (Intel Corporation, Santa Clara, CA, USA). The platform’s LiDAR and manipulator do not contribute to risk or sparse recovery (Figure 4).

**Figure 5:**
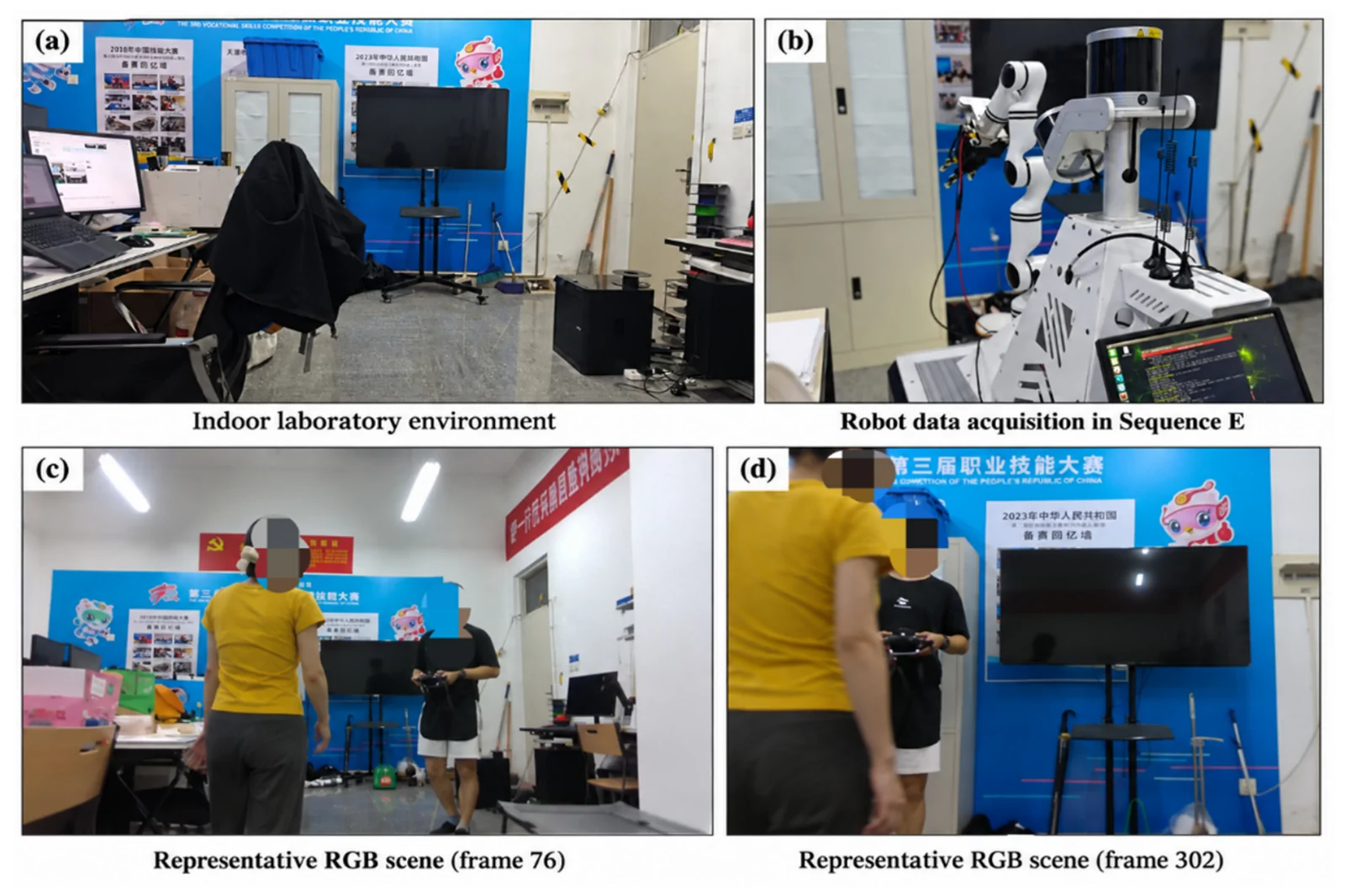
Indoor experimental environment, mobile-robot data acquisition, and representative scenes from Sequence E. The Chinese text visible on background banners and posters is incidental environmental signage and is unrelated to the experimental methodology or results.

Sequence E comprises 320 paired RGB-D frames with robot and human motion (Figure 5). RGB enters LingBot-Map; registered D435i depth is used only as an offline sensor-depth reference or for selected sparse anchors, never for M_t_. It is not independent high-accuracy ground truth.

The separate Bonn geometry experiment reconstructs all methods with shared ground-truth poses to isolate depth-derived geometry differences, not camera-pose accuracy (Table 2).

### 4.2. Comparison Methods and Budget Protocol

For RQ1, M_t_ is compared with median native confidence (ConfMedian) and its tenth percentile (ConfP10). Lower confidence implies higher priority; confidence scores are not assumed to be calibrated errors.

RQ2 compares Full-Reference Global (single-scale, complete-reference diagnostic), Sparse Global (single-scale, 2% anchors), and Spatial (global-referenced local correction on exactly the same anchors). Sparse Global versus Spatial isolates the local operator.

RQ3 compares M-TopK, ConfMedian-TopK, ConfP10-TopK, Random, Periodic, and offline Oracle-Utility. All receive equal frame and anchor budgets; Oracle ranks by measured recovery utility and is not deployable.

Two frame-budget protocols are evaluated. The retrospective reference-normalized diagnostic considers budgets of 2%, 5%, 8%, 10%, 15%, 20%, 30%, 40%, and 50%; it uses complete-reference global-scale normalization to score unselected frames and thus isolates frame ranking under a common scale-aligned metric. The historical sparse-scale propagation protocol instead uses a common initial 2% sparse observation, charged to the 20% frame budget, and applies the most recent previously measured global scale on unselected frames. Selected frames in both protocols use 2% depth anchors. The protocols are reported separately because their unselected-frame information access and output definitions differ.

The intervention set in Section 3.3 is defined over the multi-reference-risk-valid set *T_M_*. For the budget benchmark, however, a frame must have both a valid formal risk and a valid recovery evaluation. We therefore define the common evaluation set as

(11) *T*_eval_ = *T_M_* ∩ *T_R_*

*T_R_* contains recovery-evaluable frames; intersection with *T_M_* yields the common benchmark set, not a change to the formal method definition.

### 4.3. Evaluation Metrics

#### 4.3.1. Geometric-Risk Ranking

RQ1 ranks reference-depth error after per-frame scale alignment, using predicted *D_t_*, reference *D_t_^ref^*, and their common valid region *Ω_t_^ref^* (Equation (12)).

(12) $$\begin{array}{l} s_{t}^{\mathrm{ref}}=\frac{\underset{u\in \Omega_{t}^{\mathrm{ref}}}{\mathrm{median}}D_{t}^{\mathrm{ref}}(u)}{\max\left(\underset{u\in \Omega_{t}^{\mathrm{ref}}}{\mathrm{median}}D_{t}(u),\delta \right)},\\ E_{t}^{\mathrm{ref}}=\frac{1}{\left|\Omega_{t}^{\mathrm{ref}}\right|}\sum_{u\in \Omega_{t}^{\mathrm{ref}}}\frac{\left|s_{t}^{\mathrm{ref}}D_{t}(u)-D_{t}^{\mathrm{ref}}(u)\right|}{D_{t}^{\mathrm{ref}}(u)} \end{array}$$

Registered dataset depth supplies *D_t_^ref^* for public scenes and D435i depth for Sequence *E*. Spearman ρ assesses M_t_ or confidence ranking relative to *E_t_^ref^*; lower confidence is interpreted as higher priority.

*E_t_^ref^* in Equation (12) is a ranking metric, not the full-reference-normalized held-out base error used in RQ2/RQ3; their masks and roles differ.

#### 4.3.2. Depth and 3D Geometry Recovery Quality

For RQ2, 2D depth recovery is evaluated using held-out AbsRel. Let *Ω_t_^held^* denote the valid reference-depth evaluation mask after excluding sparse anchor pixels. For a candidate depth map *D*, held-out AbsRel is

(13) $$\mathrm{AbsRel}=\frac{1}{\left|\Omega_{t}^{\mathrm{held}}\right|}\sum_{u\in \Omega_{t}^{\mathrm{held}}}\frac{\left|D(u)-D_{t}^{\mathrm{ref}}(u)\right|}{D_{t}^{\mathrm{ref}}(u)}$$

Excluding anchor pixels from *Ω_t_^held^* evaluates correction on unmeasured locations.

The complete held-out reference provides a single-scale diagnostic baseline *E_t_^base^*, while *E_t_^rec^* denotes the held-out error after local recovery. Their difference defines offline single-frame utility in Equation (14).

(14) $$U_{t}=E_{t}^{\mathrm{base}}-E_{t}^{\mathrm{rec}}$$

Positive *U_t_* denotes beneficial recovery. Correlations of *M_t_*, ConfMedian, and ConfP10 with *U_t_* assess how each ranks potential intervention benefit.

The Bonn shared-pose Leica evaluation reports both nearest-neighbor directions, symmetric Chamfer-L1, precision/recall/F-score at fixed distance thresholds, and valid-output coverage. These are distinct from 2D AbsRel and pose accuracy.

#### 4.3.3. Budget Efficiency

In the reference-normalized diagnostic, selected frames use sparse recovery and unselected frames use complete-reference scale-normalized baseline error. This isolates offline ranking, not deployable unselected-frame performance.

Under historical sparse-scale propagation, unselected frames use the latest previously measured global scale, with the initial query counted. Selected frames use matched 2% anchors. Frame rankings remain retrospective.

(15) $$G_{M/\mathrm{Random}}=\frac{E_{\mathrm{base}}-E_{M}}{E_{\mathrm{base}}-E_{\mathrm{Random}}}$$

Equation (15) defines the retrospective gain relative to Random, using a common reference-normalized Base. A gain greater than one indicates a larger diagnostic error reduction. It is undefined if Base equals Random and not a positive improvement ratio if Random is no better than Base. Neither this gain nor the reference-informed Oracle policy applies to temporally coupled historical-scale evaluation.

### 4.4. Implementation Details and Protocol Controls

All analyses use frozen LingBot-Map predictions. Risk uses lag offsets {3, 6}, stride 6, P90 relative residuals, and the arithmetic mean of two valid single-lag scores.

A trajectory audit compared exported and inverted pose conventions against reference poses on five sequences. At lag 6, median rotation errors were 0.339–0.652° for C2W versus 3.062–9.142° when inverted. This supports C2W direction, not error-free poses or shared depth–translation scale.

Sparse recovery uses a valid registered depth of 0.1–10 m and 2% uniformly sampled anchors. A 32 × 32 block uses its local scale only with ≥2 valid anchors and a local/global ratio in [0.25, 4.0]; otherwise, it uses the global estimate. Fewer than ten valid frame ratios trigger retention of original depth.

Random trial counts are defined per evaluation protocol rather than globally: the reference-normalized retrospective diagnostic averages 1000 random schedules, the eleven-scene historical-scale comparison averages 200 per scene, and the fixed-mask repeated-anchor experiment reuses 50 schedules for each seed. At a given protocol and frame budget, compared schedules use the same eligible evaluation frames, initial query rule, and number of selected frames. Anchor pixels are excluded from held-out depth scoring, and repeated schedules or seeds within a scene are not treated as independent scenes.

Public dataset depth supports 2D evaluation; Sequence E uses continuously recorded D435i depth as a sensor reference, not independent 3D ground truth. Bonn uses Leica geometry and shared GT poses. These reference types must not be pooled.

OFAT studies vary nine risk configurations (1958 targets, three scenes) and nine recovery configurations (2739 frames, five scenes, three seeds). Pose/scale controls use frozen nonzero-translation pairs. Bonn’s independent Leica study uses 966 shared-pose frames, a fixed Leica/GT-defined core with shared original-anchor exclusion, and a greater measurement budget for Full-Sensor than for 2% variants.

## 5. Results and Discussion

Results are organized by risk ranking (RQ1), matched sparse recovery (RQ2), and the two retrospective allocation protocols (RQ3).

### 5.1. Effectiveness of Multi-Reference Geometric Risk

Risk M_t_ uses only backbone outputs; registered depth enters the offline reference-error calculation, not risk estimation.

Across 11 public scenes, median Spearman ρ (M_t_, scale-aligned depth error) is 0.635, including Bonn crowd 0.882, moving_box 0.750, and TUM walking_rpy 0.739. Stairs seq01 is a negative case (ρ ≈ 0.001), showing that risk is not calibrated frame error.

The frozen Stairs seq01 audit retained 314 eligible frames: final dual-reference M_t_ correlated poorly with reference error (ρ = 0.001445) but more with single-frame recovery utility (ρ = 0.291146), assessed on distinct reference domains. We correlated the same M_t_ with lag-specific relative rotation and source-normalized valid overlap, not with the individual lag P90 scores. Overlap is valid warped/current matches divided by 8560 stride-6 source-grid locations, not target-image coverage.

For k = 3/6, correlations of final M_t_ with predicted rotation are 0.509/0.552 and with source-normalized overlap −0.473/−0.585. Individual lag P90 scores correlate near zero with reference error (−0.0008/−0.0015). A circular 16-frame moving-block bootstrap (1000 draws) yields a descriptive 95% range [−0.426,0.397] for ρ(M_t_,error), not evidence of zero population correlation or a unique physical cause.

Sequence E has 314 risk-valid frames among 320 pairs, with ρ(M_t_, scale-aligned registered-depth error) = 0.556. Temporal dependence precludes treating successive frames as independent significance replicates. The registered depth is an evaluation reference, not a risk input.

Lag 3 and lag 6 provide complementary baselines; M_t_ is defined only when both comparisons meet minimum support. Missing-lag risks are not imputed. Table 3 summarizes the frame-level ranking performance of Multi-Reference Geometric Risk across 11 public scenes and compares its cross-scene median correlations with native-confidence baselines.

Across 11 scenes, median error-ranking correlations for M_t_, ConfMedian, and ConfP10 are 0.635, 0.204, and 0.466; the corresponding recovery-utility correlations are 0.438, 0.136, and 0.482. Thus, M_t_ provides the strongest median error ranking, whereas ConfP10 ranks median recovery utility slightly better; neither signal universally dominates.

**Figure 6:**
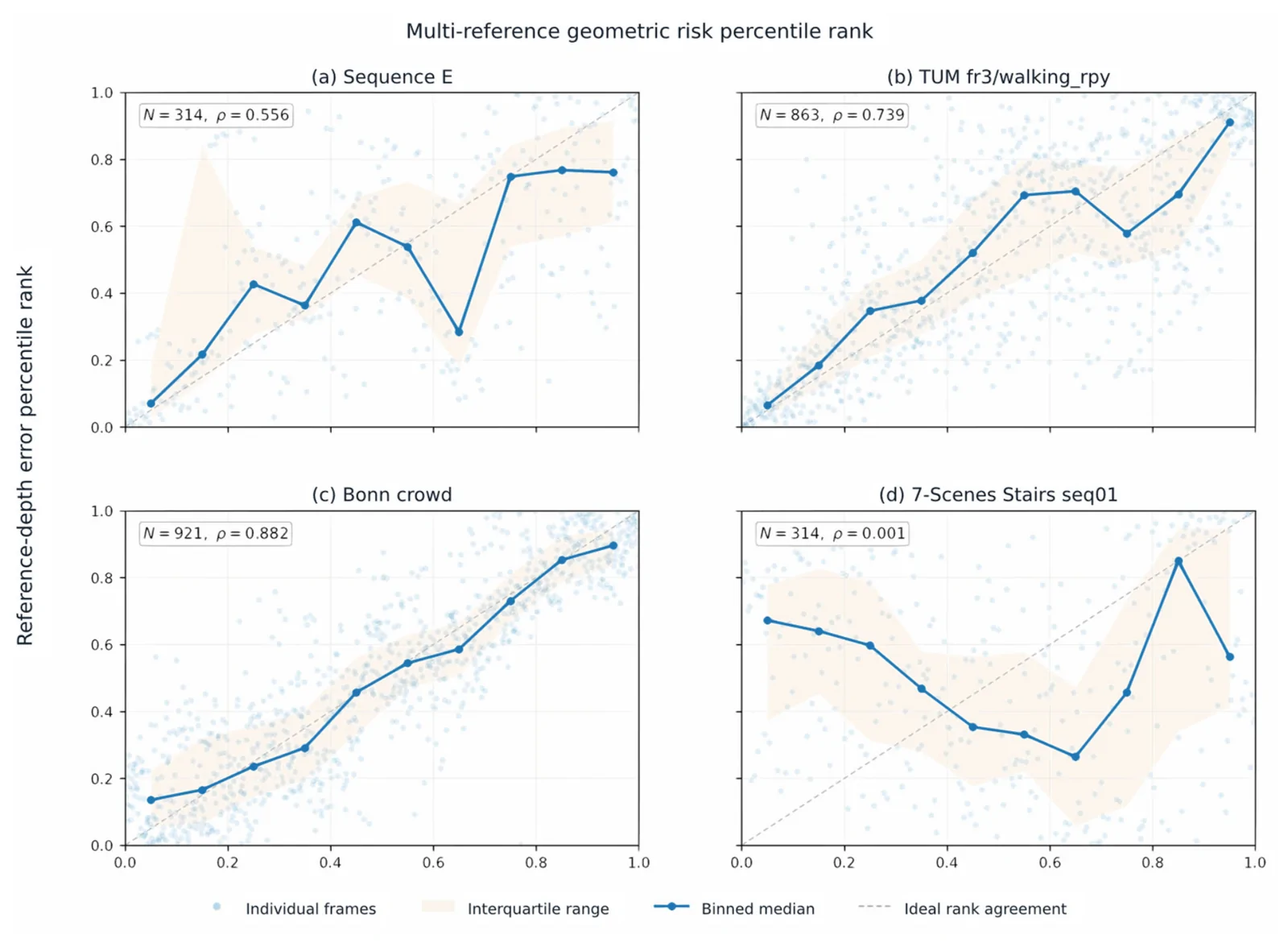
Percentile-rank relationship between Multi-Reference Geometric Risk and reference-depth error for Sequence E, TUM walking_rpy, Bonn crowd, and 7-Scenes Stairs seq01.

Cross-view inconsistency can reveal local deformation but miss geometrically consistent bias. It can also reflect pose, motion, and occlusion effects rather than pure depth error. Figure 6 illustrates the percentile-rank relationships between geometric risk and reference-depth error across four representative sequences.

### 5.2. Sparse Geometric Anchoring and Local Recovery

RQ2 isolates local correction by comparing Spatial and Sparse Global on the same 2% anchors, alongside Full-Reference Global as a single-scale diagnostic.

**Figure 7:**
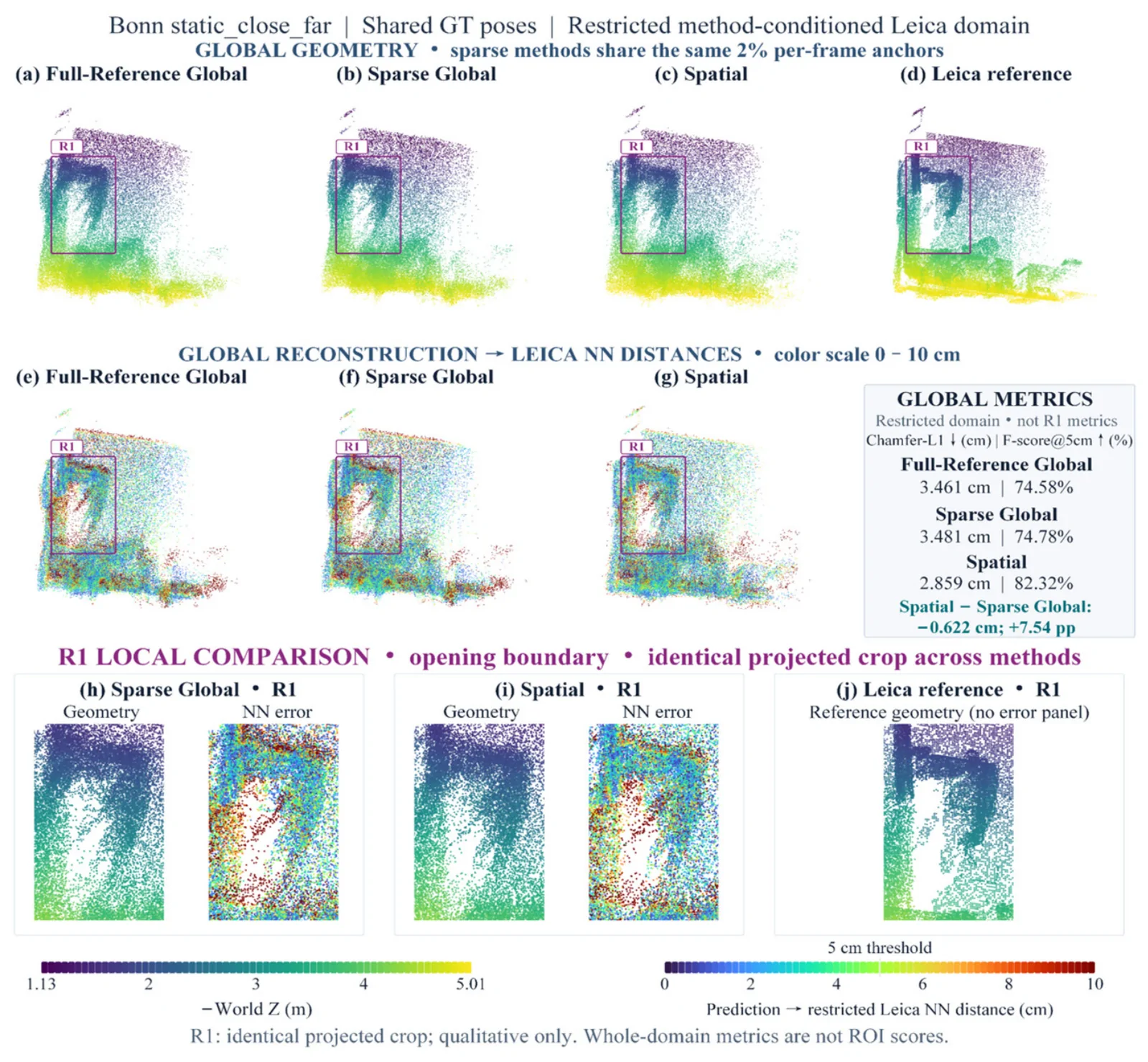
Bonn static_close_far 3D geometry in the restricted, method-conditioned Leica domain (966 shared-GT-pose frames; 98,836 reference points). (**a**–**d**) Full-Reference Global, Sparse Global, Spatial, and restricted Leica geometry; (**e**–**g**) corresponding one-way Leica NN-error views; (**h**–**j**) matched opening-boundary R1 details. Both sparse methods use identical 2% anchors per frame. Chamfer-L1 and core summarize the full restricted domain, not R1. A separate frozen Leica/GT-defined core with common original-anchor exclusion is evaluated later in Section 5.4.2. Residual high-error points remain in the full-scene Spatial NN-error view.

In the restricted, method-conditioned Bonn Leica domain, Chamfer-L1 is 3.461/3.481/2.859 cm and F-score@5cm is 74.58/74.78/82.32% for Full-Reference Global/Sparse Global/Spatial (Figure 7). Across the same 966 GT-pose frames, eligible stride-4 pixels require valid registered sensor depth and valid outputs from all three methods. Their sensor-derived points must lie within 5 cm of Leica (implemented as a nearest-neighbor distance ≤ 0.05 m), while matched Leica points require support from at least two distinct sensor-observation frames. The resulting restricted reference contains 98,836 Leica points; the historical 3D construction does not explicitly exclude sparse anchor pixels. This screening distance is distinct from the 5 cm F-score threshold. The full-domain scores are conditional on these restrictions, not independent full-scene accuracy. R1 zooms show qualitative geometry and one-way NN error, not local Chamfer/F-score; residual high-error Spatial points remain visible.

**Figure 8:**
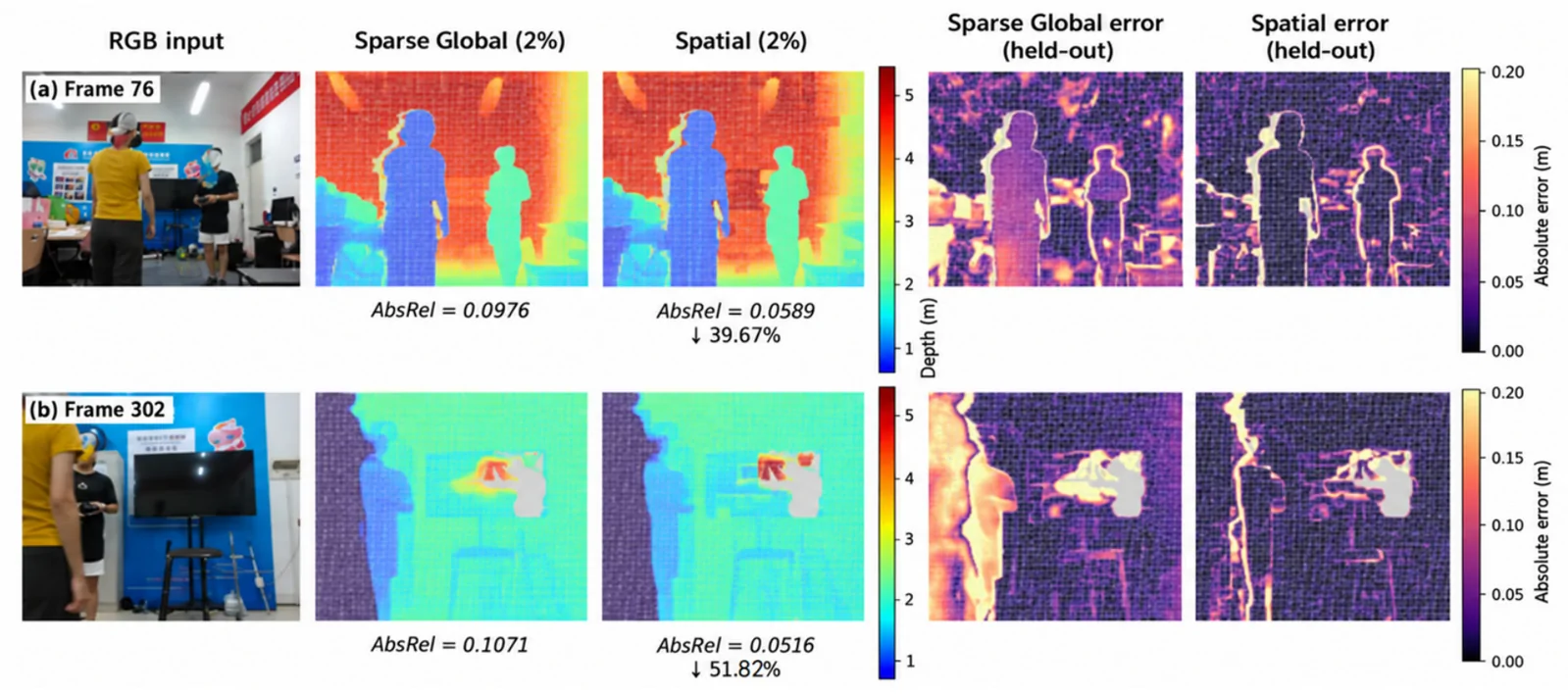
Representative sparse depth recovery results on the real-robot Sequence E (frames 76 and 302). From left to right, the panels show RGB images, depth maps recovered by Sparse Global and Spatial using identical 2% sparse anchors, and their corresponding pixel-wise absolute depth errors relative to the registered D435i depth reference. Error maps are displayed in meters over valid held-out pixels, whereas the annotated AbsRel values represent dimensionless relative depth errors. The downward arrows (↓) indicate the percentage reduction in held-out AbsRel relative to Sparse Global.

On Sequence E, Full-Reference Global, Sparse Global, and Spatial achieve mean held-out AbsRel values of 0.104983, 0.101283, and 0.060068, respectively (Table 4b). With identical 2% sparse depth anchors, Spatial reduces the mean held-out AbsRel by **40.69%** compared with Sparse Global. Figure 8 presents the representative depth recovery results and spatial error distributions for two selected frames.

Full-Reference Global uses complete reference information but fits only one frame scale, so it cannot remove local scale deformation. Its comparison with Spatial does not imply that sparse data contain more information; the local model has greater spatial flexibility (Table 4).

Figure 7 and Table 4 report results for a restricted, method-conditioned evaluation domain. A separate evaluation described in Section 5.4.2 uses the same 966 real frame IDs and shared GT poses, but a different evaluation domain. The former applies sensor- and method-conditioned verification (≤5 cm sensor-to-Leica distance, at least two distinct sensor frames of support) without explicit 3D anchor exclusion. The latter uses a fixed Leica/GT-visible core and excludes the same original sparse anchors for every method, without intersecting output-validity masks or applying a sensor-to-Leica error gate. Their 98,836- and 183,503-point references, and hence their absolute Chamfer/F-score values, are not interchangeable.

### 5.3. Intervention Allocation Under a Limited Frame Budget

Under the retrospective reference-normalized protocol with a 20% frame budget, M-TopK achieves a gain ratio above one relative to Random across all 11 public scenes (median 1.510; Table 5), including Bonn moving_box (2.799), crowd (1.939), and Stairs seq04 (1.820). These results indicate the diagnostic value of geometric risk for retrospective frame ranking. However, they do not establish consistent improvements under historical sparse-scale propagation or a causal online measurement-selection policy. The small allocation gains and their temporal sensitivity are further examined below.

Sequence E reference-normalized protocol uses 314 frames and 63 queries: Base 0.104522, M-TopK 0.093608, Random 0.095585, Periodic 0.095664, ConfP10 0.094859, ConfMedian 0.096455, and Oracle-Utility 0.090272 (Table 5b). Random averages 1000 schedules; M/Random gain is 1.221. These are diagnostic, not deployable outputs.

Paired mean AbsRel differences (M minus comparator) are −0.001977 against Random, −0.001251 against ConfP10, and −0.002056 against Periodic, representing 2.07%, 1.32%, and 2.15% lower mean error. The gain ratio 1.221 refers to relative reductions from Base, not 22.1% lower AbsRel; small differences require uncertainty analysis.

Temporal sensitivity uses paired per-frame difference d_t_ = e_t_(M) − e_t_(C) on the same 314 frames; Random contributes the per-frame mean over 1000 frozen schedules. Non-circular moving blocks of length 8, 16, or 32 are sampled uniformly by valid start index, concatenated and truncated to 314 indices. Identical sampled indices weight both methods; normalized frame occurrence counts define the mean. Each of 10,000 repetitions contributes a paired mean difference, whose 2.5th/97.5th percentiles give Table 5c ranges. Selections, anchors, and 63 queries remain fixed. These conditional single-video, time-reweighting ranges, all crossing zero, are not cross-environment confidence intervals or evidence of equivalence.

The 0.104983 Full-Reference Global error in Section 5.2 uses a different recovery-evaluable set from the 314-frame Base 0.104522 in Section 5.3; the two protocol means must not be compared directly.

**Figure 9:**
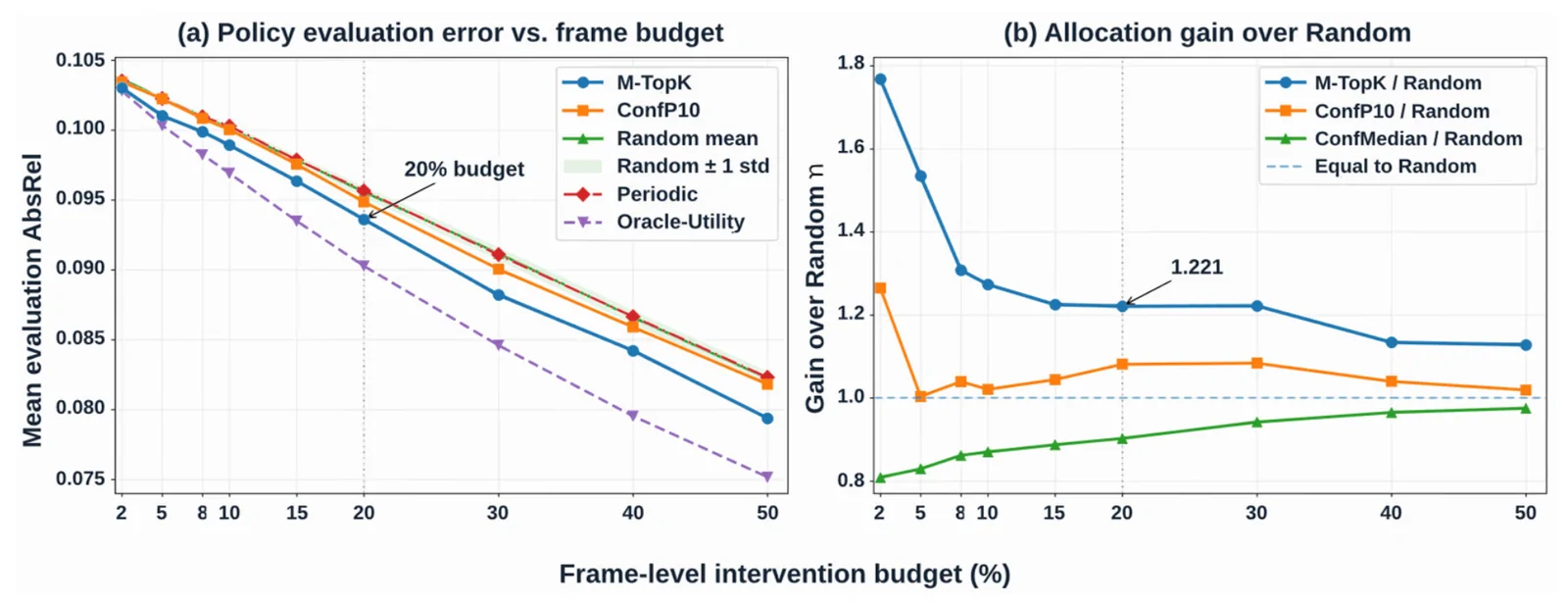
Reference-normalized retrospective frame-allocation gains on Sequence E across frame-level query budgets; not an online measurement-cost evaluation.

All-frame recovery reaches 0.059925 mean AbsRel in this reference-normalized diagnostic; 20% M-TopK captures 24.47% of the maximum observed Base-to-all-frame reduction. This fraction is diagnostic, not saved sensor cost. Figure 9 illustrates the reference-normalized allocation error and the gain over Random across different frame-level query budgets on Sequence E.

#### Historical Sparse-Scale Propagation and Matched Recovery Evaluation

Historical sparse-scale propagation uses the fixed eligible frame set starting at frame 8; that first 2% measurement counts toward K = floor(0.20N), with later queries updating the carried scale. Unselected frames retain the latest measured scale. Evaluation is retrospective, not a causal scheduler.

In the 11-scene Last Valid Spatial (L-S) historical-scale evaluation, macro-mean AbsRel is 0.068824 for M-TopK, 0.063992 for Random, 0.063522 for Periodic, 0.067282 for ConfP10, and 0.072920 for ConfMedian (Table 6). Thus M-TopK is not universally better under temporal scale propagation. The M − Random macro difference is +0.004832 AbsRel; the scene-resampling sensitivity range [−0.000591, +0.014161] measures dependence on the particular 11-scene composition, not uncertainty over independent environments.

Under matched anchors, Spatial improves on Sparse Global in 55/55 scene–policy means and 200/200 fixed-mask scene–policy–seed means. However, under the separate 312-frame historical-scale protocol, Sequence E ten-seed M-TopK minus Random is +0.000218 AbsRel (SD 0.000190; M wins 1/10); versus Periodic, it is +0.000153 (2/10), and versus ConfP10 +0.000024 (2/10). These are within-sequence repeated-anchor comparisons, not independent environments or formal confidence intervals.

On the separate Sequence E 314-frame/K = 63 diagnostic, ten matched anchor realizations favor M over Random, ConfP10, and Periodic in all ten. Mean M − Random is −0.002054 AbsRel (seed SD 0.000055). Seeds alter both anchors and complementary held-out masks, so this tests the realization stability, not the time or cross-scene generalization; it is distinct from 312-frame/K = 62 historical-scale repeats.

On Sequence E, Last Valid and log-scale EMA (β = 0.5) share 312 frames, K = 62 counted queries including an initial frame-8 sparse measurement, and matched anchors/masks. Both states initialize directly at the measured frame-8 global scale. After each subsequently selected valid measurement, EMA sets the new log-scale state to the equal-weight average of its previous log state and the newly measured log global scale. Selected frames use their current sparse Global or Spatial correction; the carried EMA state scales, only later, unselected predictions. For M-TopK, Last Valid/EMA AbsRel is 0.102300/0.103216 (Global) and 0.092852/0.093768 (Spatial); for Periodic, it is 0.100855/0.100977 and 0.092821/0.092942; for Random (1000 schedules), 0.100944/0.101023 and 0.092797/0.092876. Under this specific β and protocol, EMA does not outperform Last Valid; this does not assess all temporal-fusion methods.

Component CPU timings use an i9-9900K, nine scene/frame fixtures, five warm-ups, and 30 single-core repetitions per fixture/component. Pooled median times are 1.911–2.660 ms (two-lag risk), 0.371–0.580 ms (2% anchor/Global), 5.527–9.787 ms (Spatial), and 0.101–0.226 ms (Last Valid). In-memory timings exclude backbone inference, transmission, and reference scoring; they are not end-to-end latency.

### 5.4. Parameter Sensitivity and Independent Geometry Evaluation

#### 5.4.1. Predeclared Parameter and Geometric Controls

Risk OFAT compares lag pairs {1, 3}/{3, 6}/{6, 12}, projection strides 3/6/12, residual quantiles P80/P90/P95, and minimum valid-overlap supports 100/200/400. The frozen depth and poses, evaluation frame mapping, and reference-depth error definition are held fixed for these risk comparisons. On walking_rpy, walking_xyz, and Heads, stride variants retain near-identical rankings (ρ = 0.995–1.000), whereas lag and quantile changes more strongly affect ranking and Top-K membership. Raising the minimum-overlap threshold to 200/400 does not change the tested valid sample, which does not establish behavior under low-overlap conditions. The separate sparse-recovery OFAT uses fixed held-out pixels/masks and predefined anchor seeds.

A separate median-scale ratio-gate OFAT compares (0.05, 20), default (0.1, 10), (0.2, 5), and (0.5, 2), holding the warping, support rules and full P90 residual domain fixed. Default reproduces 1870 outputs to max difference 2.22 × 10^−16^; gate variants preserve the 314/314/314/863 eligible frames of Stairs seq01/seq04/Heads/TUM walking_rpy.

Gate ranking correlations with default are ≥0.999990889, and shared-set 20% Top-K subsets are identical (Jaccard = 1). This check uses floor(0.20N), hence K = 62 for each 314-frame scene and K = 172 for TUM; it does not replace Section 5.3 outcomes. For Stairs seq01 under the narrowest gate, the correlation between geometric risk and reference error remains ρ = 0.001371. Stability under these gates is not parameter optimality or gauge correction.

Across five scenes and three fixed anchor seeds, recovery gate [0.25, 4] yields mean Spatial AbsRel 0.04909, compared with 0.05432 for [0.5, 2] and 0.04695 for [0.125, 8]. Minimum local-anchor support 1/2/4 yields 0.04879/0.04909/0.05030; Table 7 reports density and block-size controls. All 135 scene–seed–configuration means favor Spatial over Global, though three individual frames favor Global. The default is conservative, not empirically optimal.

Frozen pose/scale controls cover 3872 nonzero-translation frame pairs (1936 targets; k∈{3, 6}) from TUM walking_rpy, walking_xyz, and Heads. Reference poses were mapped by a frozen Sim(3) fit using predicted and reference camera centers; the alignment is not predictor-independent. On the shared 1936-target subset, mean M_t_ changes under pose substitution by −0.004657 (walking_rpy) and +0.003651 (walking_xyz). With intrinsics, rotations and risk computation fixed, separate 0.5×/2× depth or translation scaling shifts projection by 2.02–25.98 px (scene–configuration means) and changes risk ranking (Spearman 0.268–0.985). Joint depth–translation scaling leaves projection, median scale, and P90 unchanged in 7744 stored comparisons. P90 uses each condition’s finite-overlap domain, so this demonstrates sensitivity, not fixed-domain causal decomposition or verified historical common gauge.

#### 5.4.2. Full-Depth Reference and Bonn Leica Geometry

On Sequence E, comparing Full-Sensor depth with its own D435i-derived reference yields zero held-out error by construction and uses a larger depth budget. The Bonn static_close_far 3D comparison instead uses three methods (Full-Sensor, Sparse Global, Spatial) on the same 966 GT-pose frames; both sparse variants receive 2% anchors on every frame, not a 20% temporal frame budget. Table 8 employs a separately frozen Leica/GT-defined core: fixed ROI, valid GT-view projections and z-buffer visibility, support from at least two GT views, and a shared stride-4 evaluation mask excluding the original sparse anchors. The anchor mask was generated from prediction- and sensor-valid candidates; thus, this core is not wholly independent of their sampling information. It does not use a common intersection of method-output validities or a sensor-to-Leica distance gate. After 2 cm voxel aggregation the frozen Leica reference has 183,503 points. Each method’s invalid outputs affect its own cloud and reported coverage, not the shared reference; no method-specific ICP/Sim(3) alignment is applied.

In this separately frozen Leica/GT-defined domain (Table 8), Spatial versus Sparse Global reduces completeness distance 7.68→6.17 cm and raises Recall@5cm 67.64→71.59% but increases accuracy distance 16.57→19.03 cm and Chamfer-L1 12.13→12.60 cm while reducing F-score@5cm 45.62→40.95%. At 2 cm, Spatial does not surpass Sparse Global in recall. This single shared-pose scene demonstrates an accuracy–completeness trade-off, not universal 3D gains. The different method rankings in Table 4a and Table 8 further highlight the importance of evaluation-domain selection. Table 4a reports performance within a restricted domain conditioned on sensor–Leica agreement and common method validity, whereas Table 8 evaluates a separately frozen Leica/GT-defined domain without method-validity intersection or sensor-to-Leica error filtering, while retaining a common original-anchor exclusion. Consequently, the favorable restricted-domain results do not establish general 3D superiority. In the frozen domain, Spatial improves recall but reduces precision and F-score and increases Chamfer-L1 relative to Sparse Global. These observations demonstrate an evaluation-dependent accuracy–completeness trade-off rather than identifying a unique physical cause. Moreover, the held-out 2D depth improvements on Sequence E should not be interpreted as guaranteed 3D reconstruction gains on Bonn.

### 5.5. Discussion and Limitations

Risk is an uncalibrated cross-view discrepancy signal (public median error-ranking ρ = 0.635), affected by depth, pose, motion, occlusion, and gauge consistency. Stairs seq01 (ρ ≈ 0) is a negative case, and native confidence can rank recovery utility better in some scenes.

Stairs motion/overlap covary with final risk but cannot explain its near-zero error ranking; the four gate controls do not remove the failure. Time-block intervals are descriptive for temporally dependent frames and do not identify a unique physical cause or imply cross-scene generalization.

Matched-anchor Spatial improves depth error, while the independent Bonn Leica evaluation shows higher completeness/recall but worse accuracy/Chamfer/F-score than Global. Thus, local depth refinement does not establish uniform independent 3D superiority.

Historical sparse-scale allocation is vulnerable to stale measurements: for TUM walking_rpy frames 533–546, M-TopK carries frame-443 scale and averages AbsRel 0.384732 versus 0.028463 for Periodic. This demonstrates protocol-dependent temporal failure, not an identified physical cause.

Sequence E’s reference-normalized allocation benefits are small (M − Random −0.001977; M − ConfP10 −0.001251) and positive across ten matched anchor realizations, but time-block ranges cross zero. In 11-scene historical-scale L-S, M − Random/Periodic macro differences instead favor comparators (+0.004832/+0.005301). Ranking benefits are therefore protocol- and scene-dependent, not stable general gains.

The frame budget models selectively use already recorded D435i depth, not acquisition, energy, or transmission savings. Full-window Top-K is retrospective, and component CPU timings exclude full backbone and end-to-end runtime.

The backbone is fixed, Sequence E reference and anchors share the D435i source, and Bonn Leica is a single shared-pose scene. Five-sequence pose audits support C2W convention but not universally correct poses or shared depth–translation scale; post-warp alignment cannot restore displaced correspondences. These limits preclude causal online or general 3D deployment claims.

## 6. Conclusions

We combine training-free multi-reference frame-risk estimation with conservative 2% sparse depth anchoring of frozen LingBot-Map outputs. Retrospective allocation and sparse recovery are evaluated separately; camera poses, weights, and historical state are unchanged.

Across 11 public scenes, the median risk–error Spearman correlation is 0.635; the single real-robot Sequence E yields ρ = 0.556. With matched anchors, Spatial reduces Sequence E held-out AbsRel by 40.69% against Sparse Global and improves mean depth error in 55 scene–policy and 200 fixed-mask comparisons. In the Bonn evaluation on the frozen Leica/GT-defined core, Spatial improves completeness and recall but yields higher Chamfer-L1 and lower F-score than Sparse Global. The small retrospective allocation gains remain temporally uncertain.

Risk complements model-native confidence and remains sensitive to pose/scale mismatch. The approach is a controlled output-level depth intervention and not a causal sensor scheduler or evidence of measured sensing-cost reductions.

## Figures and Tables

**Table 1:** Comparison of problem settings and intervention mechanisms with representative related methods.

Method	Main Objective	Reliability/Selection Signal	External Geometry	Intervention Granularity	Model/State Modification
Test3R [15]	Test-time reconstruction adaptation	Geometric consistency	No	Reconstruction/model level	Yes
TTT3R [16]	Long-sequence test-time adaptation	Alignment confidence	No	Streaming-state level	Yes
Trust3R [17]	Reconstruction uncertainty estimation	Evidential uncertainty	No	Point level	Extra uncertainty modeling
Region-aware [25]	Sparse-depth scale adaptation	—	Sparse depth	Region level	No
AREA3D [26]	Active 3D reconstruction	Active selection criterion	Task-dependent sensing	Future-view level	No
ProbeMDE [27]	Active depth measurement	Prediction uncertainty	Sparse measurements	Within-frame level	No
Motion-Aware LingBot-Map [13]	Streaming context adaptation	Motion state	No	Keyframe/context level	Context management
Ours	Budgeted external-geometry intervention	Multi-Reference Geometric Risk	Sparse geometry	Frame level + selected-frame recovery	No

**Table 2:** Datasets, scenes, and their roles in the experimental evaluation.

Dataset	Scenes/Sequences	No.	Main Evaluation Role	Reference Geometry
TUM RGB-D [28]	walking_rpy; walking_xyz; sitting_static; sitting_xyz	4	Risk ranking; budget allocation	Dataset depth
Bonn Dynamic RGB-D [29]	person_tracking; crowd; moving_box; static_close_far	4	Risk ranking; budget allocation; 3D recovery	Depth + shared GT poses
7-Scenes [30]	Heads; Stairs seq01; Stairs seq04	3	Risk ranking; budget allocation	Dataset depth
Sequence E	Indoor mobile-robot sequence	1/320 frames	Risk validation; 2D recovery; budget allocation	Registered D435i depth

**Table 3:** Frame-level ranking performance of Multi-Reference Geometric Risk and native-confidence baselines. (**a**) Multi-Reference Geometric Risk across 11 public scenes. (**b**) Cross-scene median correlation of the evaluated frame-level reliability signals.

(**a**)			
**Dataset**	**Scene**	**ρ(M_t_, Et^ref^) ↑**	**ρ(Mt, Ut) ↑**
TUM	walking_rpy	0.739	0.621
TUM	walking_xyz	0.635	0.419
TUM	sitting_static	0.47	0.313
TUM	sitting_xyz	0.414	0.461
Bonn	person_tracking	0.675	0.438
Bonn	crowd	0.882	0.682
Bonn	moving_box	0.75	0.743
Bonn	static_close_far	0.626	0.121
7-Scenes	Heads	0.421	0.279
7-Scenes	Stairs seq01	0.001	0.291
7-Scenes	Stairs seq04	0.751	0.703
Median	—	0.635	0.438
(**b**)			
**Signal**	**Median ρ(·, Et^ref^) ↑**	**Median ρ(·, Ut) ↑**	**Interpretation**
M_t_	0.635	0.438	Geometric risk
ConfMedian	0.204	0.136	Native confidence
ConfP10	0.466	0.482	Native confidence

Note: ↑ indicates that a higher Spearman rank correlation is preferable.

**Table 4:** Bonn geometry scores on a restricted, method-conditioned verified domain and Sequence E held-out depth recovery. The restricted Bonn scores are descriptive within that domain and are not directly comparable with the separately frozen Leica/GT-defined core with common original-anchor exclusion described in Section 5.4.2. (**a**) Bonn shared-GT-pose geometry-only evaluation. (**b**) Sequence E held-outcovery.

(**a**)			
**Method**	**Chamfer-L1 Avg. ↓ (cm)**	**F5cm ↑**	
Full-Reference Global	3.461	74.58%	
Sparse Global	3.481	74.78%	
Spatial	2.859	82.32%	
(**b**)			
**Method**	**External Geometry**	**Held-Out AbsRel ↓**	**Relative Reduction**
Full-Reference Global	Full reference for scale only	0.104983	—
Sparse Global	2% anchors	0.101283	—
Spatial	2% anchors	0.060068	40.69% vs. Sparse Global

Note: ↓ indicates that lower values are better; ↑ indicates that higher values are better.

**Table 5:** Retrospective frame-allocation results under reference-normalized depth-error evaluation (20% frame budget). The baseline for an unselected frame is reference-scale normalized; these results are not historical-scale or online deployment outcomes. (**a**) Public 11-scene benchmark at a 20% frame budget. (**b**) Sequence E at a 20% frame budget. (**c**) Paired conditional 95% time-reweighting ranges for M-TopK minus comparator (AbsRel; N = 314, K = 63, fixed schedules).

(**a**)			
**Dataset**	**Scene**	**M/Random Gain ↑**	
TUM	walking_rpy	1.299	
TUM	walking_xyz	1.524	
TUM	sitting_static	1.065	
TUM	sitting_xyz	1.431	
Bonn	person_tracking	1.12	
Bonn	crowd	1.939	
Bonn	moving_box	2.799	
Bonn	static_close_far	1.689	
7-Scenes	Heads	1.002	
7-Scenes	Stairs seq01	1.51	
7-Scenes	Stairs seq04	1.82	
Median	—	1.51	
(**b**)			
**Allocation Strategy**	**Mean Evaluation AbsRel ↓**	**Remark**	
Base	0.104522	No selected-frame recovery	
M-TopK	0.093608	20% budget	
ConfMedian	0.096455	20% budget	
ConfP10	0.094859	20% budget	
Random	0.095585	1000-trial mean	
Periodic	0.095664	20% budget	
Oracle-Utility	0.090272	Offline reference	
(**c**)			
**Block Length**	**M − Random Mean**	**M − ConfP10**	**M − Periodic**
8 frames	[−0.008380, +0.003082]	[−0.009072, +0.004760]	[−0.008573, +0.002860]
16 frames	[−0.010020, +0.003578]	[−0.010381, +0.005069]	[−0.010179, +0.003339]
32 frames	[−0.012169, +0.003905]	[−0.011562, +0.004393]	[−0.012263, +0.003640]

Note: ↑ indicates that higher values are better; ↓ indicates that lower values are better.

**Table 6:** Eleven-scene held-out AbsRel under a matched 20% sparse-measurement budget with historical-scale propagation and Sparse Spatial recovery (L-S). Random values average 200 predefined schedules. Lower values indicate better depth agreement.

Dataset	Scene	M-TopK	Random	Periodic
TUM	walking_rpy	0.196882	0.147351	0.146118
TUM	walking_xyz	0.109212	0.109397	0.108941
TUM	sitting_static	0.076710	0.077229	0.077164
TUM	sitting_xyz	0.063184	0.063729	0.063549
Bonn	person_tracking	0.024990	0.023395	0.023252
Bonn	crowd	0.046406	0.046964	0.046962
Bonn	moving_box	0.050972	0.055058	0.053629
Bonn	static_close_far	0.028543	0.028042	0.027672
7-Scenes	Heads	0.048347	0.044195	0.043477
7-Scenes	Stairs seq01	0.051925	0.050985	0.050682
7-Scenes	Stairs seq04	0.059890	0.057564	0.057297
Macro mean	—	0.068824	0.063992	0.063522

**Table 7:** Selected OFAT recovery results: mean Spatial held-out AbsRel by scene (three fixed anchor seeds; smaller is better). The default is 2% anchors, 32 × 32 blocks, gate [0.25, 4], and two anchors per block.

Setting	TUM rpy	TUM Static	Bonn Box	Heads	Seq. E
Default	0.09812	0.04667	0.02207	0.01871	0.05986
Anchors 1%	0.10181	0.05002	0.02306	0.02048	0.06651
Anchors 4%	0.09679	0.04533	0.02175	0.01801	0.05752
Block 16	0.09413	0.04493	0.02042	0.01730	0.06864
Block 64	0.11199	0.06003	0.02873	0.02339	0.07056

**Table 8:** Bonn static_close_far single-sequence Leica evaluation with shared ground-truth poses (966 frames; 2 cm voxel processing and a separately frozen Leica/GT-defined core with common original-anchor exclusion). Chamfer-L1 is in cm. Precision, Recall, and F-score are percentages (%) evaluated at a 5 cm nearest-neighbor threshold; Coverage (%) is the fraction of fixed-domain evaluation pixels with valid method output, independent of that threshold and distinct from geometric recall.

Method	Chamfer ↓	Precision ↑	Recall ↑	F-Score ↑	Coverage
Full-Sensor	13.80	27.61	62.06	38.22	82.48
Sparse Global	12.13	34.41	67.64	45.62	99.96
Spatial	12.60	28.67	71.59	40.95	99.96

Note: ↓ indicates that lower values are better; ↑ indicates that higher values are better.

## Data Availability

The public datasets analyzed in this study are available from their official sources: the TUM RGB-D SLAM Dataset and Benchmark (https://cvg.cit.tum.de/data/datasets/rgbd-dataset/download, accessed on 25 August 2026), the Bonn RGB-D Dynamic Dataset (https://www.ipb.uni-bonn.de/data/rgbd-dynamic-dataset/, accessed on 25 August 2026), and the Microsoft 7-Scenes RGB-D Dataset (https://www.microsoft.com/en-us/research/project/rgb-d-dataset-7-scenes/, accessed on 29 August 2026). The specific sequences used are described in the manuscript. The self-collected Sequence E is available from the corresponding author upon reasonable request.
